# Fungal Dimorphism and Virulence: Molecular Mechanisms for Temperature Adaptation, Immune Evasion, and In Vivo Survival

**DOI:** 10.1155/2017/8491383

**Published:** 2017-05-23

**Authors:** Gregory M. Gauthier

**Affiliations:** Department of Medicine, Division of Infectious Diseases, University of Wisconsin School of Medicine & Public Health, Madison, WI, USA

## Abstract

The thermally dimorphic fungi are a unique group of fungi within the Ascomycota phylum that respond to shifts in temperature by converting between hyphae (22–25°C) and yeast (37°C). This morphologic switch, known as the phase transition, defines the biology and lifestyle of these fungi. The conversion to yeast within healthy and immunocompromised mammalian hosts is essential for virulence. In the yeast phase, the thermally dimorphic fungi upregulate genes involved with subverting host immune defenses. This review highlights the molecular mechanisms governing the phase transition and recent advances in how the phase transition promotes infection.

## 1. Introduction

The ability for fungi to switch between different morphologic forms is widespread throughout the fungal kingdom and is a fundamental part of their biology. A small subset of fungi within the Ascomycota phylum is considered dimorphic, which refers to capacity to convert between two specific morphologic forms, yeast and hyphae. These fungi are capable of infecting mammals, plants, and insects, and can be subdivided into thermal and nonthermal dimorphic fungi [1]. Thermally dimorphic fungi infect humans and other mammals such as dogs, cats, armadillos, and rodents (Table 1) [2–8]. The thermally dimorphic fungi are unique among fungal pathogens because they can infect humans with normal and impaired immune defenses. This includes the etiologic agents for blastomycosis, histoplasmosis, coccidioidomycosis, paracoccidioidomycosis, and sporotrichosis. In contrast, penicilliosis and emmonsiosis occur in persons with long-standing HIV infection that has progressed to AIDS (CD4^+^ T lymphocytes ≤ 200 cells/mm^3^) or have impaired cell-mediated immunity for other reasons (e.g., solid organ transplant) [9–11]. Nonthermal dimorphic fungi can also cause human infection (e.g., *Malassezia furfur*) [12] but are more typically phytopathogenic or entomopathogenic. For example, *Ophiostoma novo-ulmi*, the etiologic agent of Dutch elm disease, has destroyed millions of elm trees in Europe and United States [13]. The “zombie ant” fungus, *Ophiocordyceps unilateralis*, secretes metabolites to alter the behavior of infected ants [14]. This review will focus on how the morphologic switch between hyphae and yeast contributes to virulence with an emphasis on thermally dimorphic fungi relevant to human health.

## 2. The Phase Transition

The reversible morphologic transition between hyphae and yeast, which is known as the phase transition, is fundamental feature of the biology and lifestyle of the dimorphic fungi [1]. In the soil (22–25°C), these fungi grow as septate hyphae that produce conidia. Disruption of soil by human activities such as construction or natural disasters can aerosolize conidia and hyphal fragments. When inhaled into the warm lungs of a mammalian host (37°C), these infectious propagules convert into pathogenic yeast (or spherules for *Coccidioides*) to cause pneumonia [1]. Once infection is established in the lungs, the yeast (or spherules) can disseminate to other organs such as the skin, bone, or brain.

Although temperature is the predominate stimulus that influences the phase transition—hyphae at 22–25°C and yeast at 37°C, additional stimuli that impact the dimorphic switch include carbon dioxide (CO_2_) tension, exogenous cysteine, and estradiol. Elevated CO_2_ tension (5% CO_2_) is required for the arthroconidia of *Coccidioides* spp. to germinate into spherules at 37°C and for optional growth of *Histoplasma capsulatum* yeast [15, 16]. In the human lung, CO_2_ tension is approximately 150-fold higher than ambient air, which provides an optimal amount of CO_2_ for phase transition [17]. In response to an upshift in temperature, mitochondrial respiration ceases in *Histoplasma, Blastomyces,* and *Paracoccidioides* [18, 19]. To reactivate respiration and complete the morphologic switch to yeast, the uptake of exogenous cysteine is required [18, 19]. The production of 17*β*-estradiol by humans influences the morphologic shift and growth of *Coccidioides* and *Paracoccidioides*, which in turn, modulates the severity of infection in women. In the presence of 17*β*-estradiol, the growth of *Coccidioides* spherules at 37°C is accelerated, which may explain the increased risk for disseminated coccidioidomycosis in pregnant women [20, 21]. Moreover, in vitro analysis has demonstrated that *Coccidioides* spherules exhibit saturable binding of 17*β*-estradiol [21]. In contrast to *Coccidioides*, the morphologic switch from hyphae or conidia to yeast in *Paracoccidioides* is blocked by 17*β*-estradiol [22, 23]. In a murine model of pulmonary infection, the conversion of conidia to yeast is impaired in female, but not in male mice [24]. In humans, the incidence of paracoccidioidomycosis is 11–30-fold higher in adult males than in adult females despite similar frequency of *Paracoccidioides* exposure. Prior to puberty, the male-to-female ratio is 1 : 1 [25].

These observations have prompted investigation to the mechanisms by which estradiol and gender influence fungal development and the host response. Gene expression microarray analysis of *P. brasiliensis* strain Pb01 demonstrated that impaired conversion to yeast at 37°C in the presence of 17*β*-estradiol reduced the transcription of genes involved with cell signaling (small GTPase RhoA, palmitoyltransferase), heat shock (*HSP40, HSP70,* and *HSP90*), chitin synthesis (chitin synthase), and glucan remodeling (*β*-1,3-glucan synthase, *α*-1,3-glucan synthase) [25]. When stimulated with paracoccin, a lectin-binding protein with chitinase activity, female mice exhibit a stronger Th1 cytokine response with increased production of tumor necrosis factor alpha (TNF-*α*), interferon gamma (INF-*γ*), and interleukin 12 (IL-12), along with increased macrophage fungicidal activity when compared to male mice [26]. Following oophorectomy and treatment with testosterone, the cytokine response shifted from Th1 to Th2 in female mice. Castration of male mice coupled with estradiol therapy favored a Th1 cytokine response instead of a Th2 cytokine response [26]. Collectively, these findings highlight the importance of sex steroid hormones and gender on fungal development and host susceptibility.

## 3. Yeast-Phase Virulence Factors and Subversion of Host Immune Defenses

Once inhaled into the lungs, conidia are ingested by macrophages, where they germinate into yeast (or spherules for *Coccidioides*) and replicate. *Histoplasma capsulatum*, *Coccidioides immitis and posadasii*, *Sporothrix schenckii*, *Paracoccidioides brasiliensis* and *lutzii*, and *Talaromyces marneffei* replicate inside and outside of innate immune cells [27–31]. Traditionally, *Blastomyces* spp. were thought to be exclusively extracellular; however, recent research demonstrates that *B. dermatitidis* conidia ingested by macrophages survive and convert to yeast [32].

During the phase transition, the thermally dimorphic fungi upregulate yeast-phase specific genes including *Blastomyces adhesion-1* (*BAD-1*), calcium-binding protein-1 (*CBP1*), yeast-phase specific-3 (*YPS3*), and spherule outer wall glycoprotein (*SOWgp*) to actively subvert host immune defenses. *B. dermatitidis* and *B. gilchristii* express BAD1 (formerly WI-1), a 120 kDA secreted, multifunctional protein serves as an adhesion and immune evasin [33–38]. Secreted BAD1 binds back to the yeast cell surface via interactions with chitin and also remains soluble in the extracellular milieu [33–35]. Cell surface-bound BAD1 binds yeast to host cells via complement receptors (CR3, CD14) and heparan sulfate to promote yeast cell adhesion to host cells. BAD-1 bound to the yeast cell surface inhibits production of TNF-*α* by macrophages and neutrophils in a transforming growth factor-*β* (TGF-*β*) dependent manner [33, 36–38]. In contrast, soluble BAD-1 blocks TNF-*α* production independent of TGF-*β* [36]. TNF-*α* is a critical cytokine for proper host defense against the dimorphic fungi. Neutralization of TNF-*α* in a murine model of infection results in progressive pulmonary blastomycosis [37]. Moreover, in 2008, the Food and Drug Administration (FDA) issued a warning of increased risk for histoplasmosis, blastomycosis, and coccidioidomycosis for persons on TNF-*α* inhibitors for treatment of autoimmune disorders (e.g., rheumatoid arthritis and Crohn's disease) [39]. In addition to affecting TNF-*α* production, BAD1 also impairs the adaptive immune response by inhibiting the activation of CD4+ T lymphocytes, which decreases IL-17 and INF-*γ* production [33]. The adhesion and immunomodulatory activities of BAD1 are essential for *Blastomyces* pathogenesis. Deletion of *BAD1* renders *Blastomyces* yeast avirulent in murine model of pulmonary infection [40]. In addition to BAD1, *Blastomyces dermatitidis* secretes a dipeptidyl-peptidase IVA (DppIVA) to modulate host immunity. DppIV is a serine protease that cleaves GM-CSF, a potent cytokine that activates macrophages and neutrophils to kill fungi [41]. Silencing DppIVA by RNA interference (RNAi) reduces survival of *B. dermatitidis* yeast cocultured with GM-CSF-activated macrophages and neutrophils [41]. Moreover, DppIVA-RNAi strains have attenuated virulence during pulmonary infection [41]. In contrast to *B. dermatitidis*, *H. capsulatum* DppIVA is not detected extracellularly and does not contribute to virulence [42, 43].

Analogous to BAD1, *Coccidioides* SOWgp is localized to the spherule cell surface and an important virulence factor. SOWgp facilitates binding of spherules to host extracellular matrix (ECM) proteins including laminin, fibronectin, and collagen [44]. Deletion of SOWgp (*SOWgp*∆) in *Coccidioides* impairs spherule adherence to ECM proteins and results in attenuated virulence in a murine model of pulmonary infection [44].

In *H. capsulatum*, *CBP1* is a secreted virulence factor that promotes intracellular replication of yeast [45, 46]. CBP1 binds calcium, exists as homodimer, is resistant to protease degradation, and is structurally related to a group of membrane lipid-binding proteins known as saposins [47, 48]. CBP1 secreted by intracellular *H. capsulatum* yeast induces macrophage apoptosis and lysis by inducing transcription of host cell caspases, transcription factors (*NUPR1/*p8, *TRB3*), and genes involved with endoplasmic reticulum (ER) stress [46]. Thus, macrophage lysis is an active process directed by the fungus and not due to high intracellular fungal burden. Similar to *BAD1*, *CBP1* is an essential virulence factor. *CBP1* null mutants (*CBP1*Δ) are unable to induce macrophage apoptosis and are avirulent in murine model of pulmonary infection [45, 46]. In addition to *CBP1*, *H. capsulatum* secretes *YPS3*, which binds back to chitin in the yeast cell wall and facilitates extrapulmonary dissemination to the liver and spleen [49].

During the morphologic switch from hyphae to yeast or conidia to yeast, dimorphic fungi undergo extensive remodeling of the cell wall including glucan composition. Reorganization of glucan content has the potential to impede recognition of pathogen-associated molecular patterns (PAMPs) by host immune cells. During the morphologic switch, the amount of *β*-(1,3)-glucan in the cell wall of *Blastomyces* and *Paracoccidioides* declines from ≈40% in hyphae to ≈5% in yeast [50, 51]. The reduction of *β*-(1,3)-glucan in the yeast cell wall may limit its recognition by dectin-1 on innate immune cells and mannose-binding lectins [17, 52]. In contrast, *H. capsulatum* does not reduce *β*-(1,3)-glucan in yeast cells, but rather it uses *α*-(1,3)-glucan as a “shield” to block dectin-1 recognition of *β*-(1,3)-glucan [53]. Thus, the dimorphic fungi utilize multiple strategies including secreted virulence factors and modification of the yeast cell wall to subvert host immune defenses to establish infection including in persons with intact immune systems.

The ability of thermally dimorphic fungi to subvert host immune defenses is not 100% effective. The host can mount an immune response to halt the progression of infection. Epidemiologic studies have demonstrated that ≈50% of persons exposed to *Blastomyces* spp. develop symptomatic infection, whereas ≈50% have asymptomatic or subclinical infection [54, 55]. Similarly, inhalation of *Histoplasma capsulatum*, *Coccidioides* spp., and *Paracoccidioides* spp. results in symptomatic infection in <10%, 33–50%, and <5% of healthy persons, respectively [56–58]. Intact innate and adaptive immune defenses along with the ability to “wall-off” yeast in granulomas are critical for host defense against infection. Following the conversion of conidia to yeast, dendritic cells and macrophages interact with and engulf yeast cells. Gene expression analysis of dendritic cells that have phagocytozed *P. brasiliensis* yeast demonstrated upregulation of transcripts involved with generating a protective immune response including TNF-*α*, IL-12, and chemokines (CCL22, CCL27, and CXCL10) [59]. In addition, the dectin-1 receptor was upregulated, which induces phagocytosis, generation of reactive oxygen species, and proinflammatory cytokines and chemokines in response to binding *β*-(1,3)-glucan [59, 60]. Chemokines promote leukocyte migration to sites of infection [59]. Similarly, macrophages infected with *P. brasiliensis* also induce a proinflammatory response with upregulation of TNF-*α*, chemokines (CCL21, CCL22, CXCL4, CXC11, and CXCL14), and kinases (IRAK2) [61]. These findings highlight the ability of the immune defenses to limit the impact of fungal virulence factors.

## 4. Regulation of the Phase Transition

The transition from hyphae or conidia to yeast at 37°C is essential for virulence. The discovery of a hybrid histidine kinase encoded by *DRK1* in *Blastomyces* and *Histoplasma* provided the first genetic proof that the morphologic switch to yeast is directly linked to virulence [62]. *DRK1* null (*DRK1*Δ), insertional mutants, and RNA interference- (RNAi-) silenced strains grow as hyphae at 37°C instead of yeast, fail to upregulate yeast-phase specific virulence factors such as *BAD1* and *CBP1*, and are avirulent in a murine model of infection [62]. The function of *DRK1* is conserved among the thermally dimorphic fungi. In *T. marneffei*, *DRKA* (a *DRK1* homolog) is critical for the conversion of conidia to yeast in macrophages [63]. In *Sporothrix, Paracoccidioides*, and *T. marneffei*, the transcript abundance of *DRK1* is higher in yeast (37°C) than in hyphae (25°C) [63–65]. *DRK1* is predicted to function as part of the high-osmolarity glycerol (HOG) signaling cascade, which facilitates adaptation to osmotic, oxidative, and temperature stresses [17]. Accordingly, *DRK1* transcription is also upregulated in response to osmotic stress in *Paracoccidioides* and *T. marneffei* [63, 65]. In addition to facilitating adaptation to temperature and osmotic stress, *DRK1* also influences the integrity of the cell wall [62, 63].

Regulation of the morphologic shift is complex and not limited to *DRK1*. The transcription factors encoded by *RYP1-4* (required for yeast phase) also govern the phase transition and regulate a set of yeast-phase specific genes involved in virulence at 37°C. These transcription factors are upregulated at 37°C and are conserved among dimorphic and filamentous fungi [66–68]. *RYP1* is a homolog of the master regulator *WOR1* in *C. albicans*, whereas *RYP2* and *RYP3* are part of the velvet complex, *VosA* and *VelB,* respectively. *RYP4* is a Zn(II)_2_Cys_6_ zinc binuclear cluster domain protein that is homologous to *A. nidulans FacB*; however, it does not appear to be involved acetate utilization [68]. These transcription factors form an integrated network in which they directly bind and regulate a common set of core genes including those important for virulence such as *CBP1* and *YPS3* [68]. Silencing *RYP1-4* transcription results in cells that fail to properly undergo the phase transition and grow as hyphae at 37°C [66–68].

The morphologic switch in the opposite direction, yeast to hyphae, is also important for pathogenesis. Growth as hyphae promotes survival in the environment, generation of conidia to facilitate transmission to new hosts, and genetic diversity through mating [1]. *B. dermatitidis SREB* and *H. capsulatum SRE1* encode a GATA transcription factor that governs the transition to hyphae following a drop in temperature from 37°C to 22–25°C [69–71]. *SREB* null mutants (*SREB*Δ) and *SRE1*-RNAi strains fail to complete the conversion to hyphae [69–71]. The role of this GATA transcription factor on temperature adaptation is conserved in other fungi. A homolog of *SREB* and *SRE1* in *C. neoformans*, *CIR1*, is essential for thermotolerance at 37°C [72]. In *B. dermatitidis*, the defect in the morphologic switch corresponds to a decrease in the biosynthesis of neutral lipids (ergosterol, triacylglycerol) and lipid droplets [70]. Supplementation with exogenous saturated fatty acids (palmitic acid, 16 : 0, and stearic acid, 18 : 0) partially corrected the defects in morphogenesis and lipid droplet formation [70]. This suggests that neutral lipid metabolism has to potentially influence the phase transition to hyphae at ambient temperature. *SREB* and *SRE1* also act as negative regulators of genes involved with siderophore biosynthesis and iron uptake; however, this role appears to be independent of the phase transition [69, 70]. In *H. capsulatum*, deletion of *VMA1*, which encodes a vacuolar ATPase involved with intracellular iron homeostasis, results in cells that fail to convert to hyphae at 25°C. This indicates the potential for iron metabolism not regulated by *SREB* to affect the temperature-dependent morphologic switch [73]. In *T. marneffei*, conversion to hyphae and maintenance of filamentous morphology at 25°C is governed by transcription factors encoded by *HGRA* and *TUPA*, respectively [74, 75]. In addition to transcriptional regulators, N-acetylglucosamine (GlcNAc) accelerates the conversion from yeast to hyphae in *B. dermatitidis* and *H. capsulatum* via *NGT1* and *NGT2* transmembrane transporters [76].

## 5. In Vivo Transcriptional Profiling

The use of forward genetic strategies such as insertional mutagenesis has substantively advanced the field of medical mycology as related to the thermally dimorphic fungi. This has led to the discovery of novel genes and gene networks that regulate the phase transition (e.g., *DRK1*, *RYP1-3*, and *SREB*). In the age of genome-wide association studies, an untapped reservoir for uncovering novel genes or gene networks in the dimorphic fungi is transcriptional profiling of yeast during infection. To identify genes important for pathogenicity, in vivo transcription profiling was performed for *Blastomyces dermatitidis* strain 26199 using a murine model of pulmonary infection [77, 78]. A novel, 2-step technique was developed to efficiently separate *B. dermatitidis* yeast from murine lung tissue to obtain high-quality RNA for RNA-sequencing (RNA-Seq) [77]. To identify *B. dermatitidis* genes with altered transcription independent of temperature or other conditions, the transcriptional profile of yeast isolated from mouse lungs was compared to yeast cocultured with macrophages at 37°C, yeast grown in vitro without bone marrow-derived macrophages at 37°C, and hyphae at 22°C using K-means cluster analysis [78]. This analysis identified 72 genes that were upregulated in vivo >2-fold and independent of temperature, macrophage cocultivation, and media conditions. A subset of these genes included those that encode proteins secreted into the extracellular milieu, metal cation uptake and transport, and amino acid metabolism [78].

Genes involved with zinc acquisition are upregulated by *B. dermatitidis* yeast during pulmonary infection. This includes a zincophore (*PRA1*/*ZPS1*), high-affinity zinc transporter (*ZRT1*), and low affinity zinc transporter (*ZRT2*) [78]. In *Candida albicans*, *PRA1* is secreted in the extracellular environment to bind zinc and deliver it to the fungus via its interaction with *ZRT1* at the cell surface [79]. In *C. albicans*, *Aspergillus fumigatus*, and *Ustilago maydis*, *PRA1* and *ZRT* are coregulated and syntenic. Although *PRA1* and *ZRT1* appear to be coregulated in *Blastomyces*, these genes are not syntenic. Surprisingly, *PRA1* is not well conserved among the dimorphic fungi and is absent in the genomes of *H. capsulatum, Paracoccidioides* spp., and *Emmonsia*; however, homologs are present in *Coccidioides*. In *C. albicans, PRA1* is postulated to impact pathogenesis. Deletion of *PRA1* results in mutants that have reduced ability to lyse endothelial cells under zinc-deplete conditions [79]. The impact of *PRA1* during in vivo infection has not yet been investigated.

In addition to upregulating zinc-scavenging mechanisms in vivo, *B. dermatitidis* increases the transcription of *NIC1*, which encodes a nickel transporter [78]. Nickel is required for the proper function of urease, an enzyme that catalyzes the conversion of urea to ammonia and CO_2_. Urea is found in mammalian tissues as a product of purine nucleotide catabolism [80]. In *Coccidioides*, urease is released from spherules during replication and damages tissue through production of ammonia, which alkalinizes the microenvironment [81]. Deletion of the urease gene (*URE*Δ) in *C. posadasii* results in attenuated virulence in murine model of pulmonary infection. At sites of pulmonary infection, *URE*Δ cells are unable to catabolize urea in lung tissue and fail to lower the pH (tissue pH 7.2 for *URE*Δ versus pH 7.7 for wild type). Moreover, mice infected with the null mutant exhibited a more organized immune response with well-formed granulomas encasing *URE*Δ cells [81]. In *Cryptococcus neoformans*, *NIC1* and *URE1* contribute to invasion of the brain. Deletion of either gene results in decreased ability for *NIC1*Δ and *URE1*Δ yeast cells to penetrate the central nervous system [82]. *URE1* also contributes to the pathogenesis of *Cryptococcus gattii*, which primarily causes pulmonary infection without an increased predilection for CNS invasion in animal models [83, 84]. *C. gattii URE1*Δ have attenuated virulence during pulmonary infection, reduced capacity to disseminate to the bloodstream, and impaired intracellular replication within macrophages [83].

During pulmonary infection, *B. dermatitidis* upregulates dioxygenases involved in the catabolism of amino acids [78]. This includes 4-hydroxyphenylpyruvate dioxygenase (4-HPPD, *HpdA*), homogentisate 1,2-dioxygenase (*HmgA*), indoleamine 2,3-dioxygenase (*IDO*), and cysteine dioxygenase (*CDG*). *HpdA* and *HmgA* are conserved among the dimorphic fungi and are localized on a gene cluster [85]. Although the precise role for *HpdA* and *HmgA* is not known in *B. dermatitidis*, research on *T. marneffei* has illuminated how these genes involved with tyrosine catabolism influence pathogenesis. *HpdA* and *HmgA* null mutants are hypersensitive to oxidative stress and have impaired spore germination to yeast in murine and human macrophages [85]. Inhibition of 4-HPPD activity appears to be important for the temperature-dependent morphologic shift. Chemical inhibition of 4-HPPD by NTBC (2-(2-nitro-4-trifluoromethylbenzoyl)-cyclohexane-1, 3-dione) in *T. marneffei* and *P. brasiliensis* blocks the conversion of conidia or hyphae to yeast following an increase in temperature from 25°C to 37°C [85, 86].

The role of fungal *IDO* on tryptophan degradation is poorly understood; however, tumor cells upregulate *IDO* to degrade tryptophan in the microenvironment to evade host immune cells [87]. Pulmonary infection with *H. capsulatum* and *P. brasiliensis* induces host IDO, which reduces fungal growth, inhibits Th17 T lymphocyte differentiation, and limits excessive tissue inflammation [88, 89].

In addition to cysteine dioxygenase (*CDG*), *B. dermatitidis* upregulates cysteine synthase A (*CSA*) and a sulfite efflux pump (*SSU1*) during pulmonary infection [78]. *CSA* encodes an enzyme involved with the biosynthesis of L-cysteine from acetyl-L-serine. *CDG* breaks down L-cysteine to L-cysteine sulfonic acid which can be further catabolized to pyruvate and sulfite. The accumulation sulfite is potentially toxic to cells and is secreted via an efflux pump encoded by *SSU1*. In *C. albicans*, deletion of *CDG1* and *SSU1* impairs hyphal development in the presence of cysteine and *CDG1*Δ, but not *SSU1*Δ, and attenuates virulence during murine infection [90]. In dermatophytes such as *Arthroderma benhamiae*, the catabolism of cysteine to sulfite by *CDO1* followed by efflux of sulfite into the extracellular environment by *SSU1* is postulated to promote breakdown of keratin to facilitate fungal growth [91]. *A. benhamiae CDO1* and *SSU1* null mutants have impaired ability to grow on keratin-rich substrates such as hair and nails [91]. On the basis of these data, there is potential that the breakdown of cysteine and sulfite secretion could promote the growth of *Blastomyces* yeast in skin, which is abundant in keratin and the most common site for extrapulmonary dissemination.

## 6. Conclusions

The thermally dimorphic fungi are a unique group of ascomycetes that are capable of infecting persons with intact and impaired immune defenses. Their ability to adapt to core body temperature (37°C) and transition to yeast morphology is essential for virulence. The morphologic switch to yeast is associated with the upregulation of specific virulence factors that promote adhesion to host tissues, growth in and lysis of macrophages, blunt proper cytokine responses, and impair cell-mediated immunity. The regulation of the reversible transition between hyphae and yeast requires these fungi to adapt and respond to numerous stimuli including temperature, CO_2_ tension, and sex hormones. In vivo transcriptional profiling has begun to uncover previously unrecognized genes important for propagation and virulence in the mammalian host.

## Figures and Tables

**Table 1:** Thermally dimorphic fungi pathogenic to humans and mammals.

Fungus	Clinical Disease
*Blastomyces dermatitidis* and *gilchristii*	Blastomycosis
*Histoplasma capsulatum*	Histoplasmosis
*Coccidioides immitis* and *posadasii*	Coccidioidomycosis
*Paracoccidioides brasiliensis* and *lutzii*	Paracoccidioidomycosis
*Sporothrix schenckii*	Sporotrichosis
*Talaromyces marneffei*	Penicilliosis
*Emmonsia* spp.	Emmonsiosis
*Lacazia loboi*	Lacaziosis

## References

[1] Gauthier G. M. (2015). Dimorphism in fungal pathogens of mammals, plants, and insects. *PLoS Pathogens*.

[2] Sarosi G. A., Eckman M. R., Davies S. F., Laskey W. K. (1979). Canine blastomycosis as a harbinger of human disease. *Annals of Internal Medicine*.

[3] Anderson J. L., Dieckman J. L., Reed K. D., Meece J. K. (2014). Canine blastomycosis in Wisconsin: a survey of small-animal veterinary practices. *Medical Mycology*.

[4] Brömel C., Sykes J. E. (2005). Histoplasmosis in dogs and cats. *Clinical Techniques in Small Animal Practice*.

[5] Graupmann-Kuzma A., Valentine B. A., Shubitz L. F., Dial S. M., Watrous B., Tornquist S. J. (2008). Coccidioidomycosis in dogs and cats: a review. *Journal of the American Animal Hospital Association*.

[6] Reis R. S., Almeida-Paes R., Mde Muniz M. (2009). Molecular characterization of *Sporothrix schenckii* isolates from humans and cats involved in the sporotrichosis epidemic in Rio de Janeiro, Brazil. *Memórias do Instituto Oswaldo Cruz*.

[7] Arantes T. D., Theodoro R. C., Mde Teixeria M., Sde Bosco M., Bagagli E. (2016). Environmental mapping of *Paracoccidioides* spp. in Brazil reveals new clues into genetic diversity, biogeography and wild host association. *PLoS Neglected Tropical Diseases*.

[8] Cao C., Liang L., Wang W. (2011). Common reservoirs for *Penicillium marneffei* infection in humans and rodents, China. *Emerging Infectious Diseases*.

[9] Kenyon C., Boorchis K., Corcoran C. (2013). A dimorphic fungus causing disseminated infection in South Africa. *New England Journal of Medicine*.

[10] Schwartz I. S., Govender N. P., Corcoran C. (2015). Clinical characteristics, diagnosis, management, and outcomes of disseminated emmonsiosis: a retrospective case series. *Clinical Infectious Diseases*.

[11] Le T., Wolbers M., Chi N. H. (2011). Epidemiology, seasonality, and predictors of outcome of AIDS-associated *Penicillium marneffei* infection in Ho Chi Minh City, Viet Nam. *Clinical Infectious Diseases*.

[12] Youngchim S., Nosanchuk J. D., Pornsuwan S., Kajiwara S., Vanittanakom N. (2013). The role of L-DOPA on melanization and mycelial production in *Malassezia furfur*. *PLoS One*.

[13] Nigg M., Laroche J., Landry C. R., Bernier L. (2015). RNAseq analysis highlights specific transcriptome signatures of yeast and mycelial growth phases of Dutch elm disease fungus *Ophiostoma novo-ulmi*. *G3*.

[14] Evans H. C., Elliot S. L., Hughes D. P. (2011). *Ophiocordyceps unilateralis*: a keystone species for unraveling ecosystem functioning and biodiversity of fungi in tropical forests?. *Communicative & Integrative Biology*.

[15] Klotz S. A., Drutz D. J., Huppert M., Sun S. H., DeMarsh P. L. (1984). The critical role of CO_2_ in the morphogenesis of *Coccidioides immitis* in cell-free subcutaneous chambers. *Journal of Infectious Diseases*.

[16] Pine L. (1954). Studies on the growth of *Histoplasma capsulatum*. I. Growth of the yeast phase in liquid media. *Journal of Bacteriology*.

[17] Gauthier G. M., Klein B. S. (2008). Insights into fungal morphogenesis and immune evasion: fungal conidia, when situated in mammalian lungs, may switch from mold to pathogenic yeasts or spore-forming spherules. *Microbe*.

[18] Maresca B., Lambowitz A. M., Kumar V. B., Grant G. A., Kobayashi G. S., Medof G. (1981). Role of cysteine in regulating morphogenesis and mitochondrial activity in the dimorphic fungus *Histoplasma capsulatum*. *Proceeding of the National Academy of Sciences, USA*.

[19] Medoff G., Painter A., Kobayashi G. S. (1987). Mycelial-to-yeast-phase transitions of the dimorphic fungi *Blastomyces dermatitidis* and *Paracoccidioides brasiliensis*. *Journal of Bacteriology*.

[20] Drutz D. J., Huppert M., Sun S. H., McGuire W. L. (1981). Human sex hormones stimulate the growth and maturation of *Coccidioides immitis*. *Infection and Immunity*.

[21] Powell B. L., Drutz D. J., Huppert M., Sun S. H. (1983). Relationship of progesterone- and estradiol-binding proteins in *Coccidioides immitis* to coccidioidal dissemination in pregnancy. *Infection and Immunity*.

[22] Restrepo A., Salazar M. E., Cano L. E., Stover E. P., Feldman D., Stevens D. A. (1984). Estrogens inhibit mycelium-to-yeast transformation in the fungus *Paracoccidioides brasiliensis*: implications for resistance of females to paracoccidioidomycosis. *Infection and Immunity*.

[23] Salazar M. E., Restrepo A., Stevens D. A. (1988). Inhibition by estrogens of conidium-to-yeast conversion in the fungus *Paracoccidioides brasiliensis*. *Infection and Immunity*.

[24] Aristizábal B. H., Clemons K. V., Cock A. M., Restrepo A., Stevens D. A. (2002). Experimental *Paracoccidioides brasiliensis* infection in mice: influence of the hormonal status of the host on tissue responses. *Medical Mycology*.

[25] Shankar J., Wu T. D., Clemons K. V., Monteiro J. P., Mirels L. F., Stevens D. A. (2011). Influence of 17*β*-estradiol on gene expression of *Paracoccidioides* during mycelia-to-yeast transition. *PLoS One*.

[26] Pinzan C. F., Ruas L. P., Casabona-Fortunato A. S., Carvalho F. C., Roque-Barreira M.-C. (2010). Immunologic basis for the gender differences in murine *Paracoccidioides brasiliensis* infection. *PLoS One*.

[27] Inglis D. O., Voorhies M., Hocking Murray D. R., Sil A. (2013). Comparative transcriptomics of infectious spores from the fungal pathogen *Histoplasma capsulatum* reveals a core set of transcripts that specify infectious and pathogenic states. *Eukaryotic Cell*.

[28] Beaman L., Benjamini E., Pappagianis D. (1981). Role of lymphocytes in macrophage-induced killing of *Coccidioides immitis* in vitro. *Infection and Immunity*.

[29] Mdel Jiménez P., Restrepo A., Radzioch D., Cano L. E., Garcia L. F. (2006). Importance of complement 3 and mannose receptors in phagocytosis of *Paracoccidioides brasiliensis* conidia by Nramp1 congenic macrophage cell lines. *FEMS Immunology and Medical Microbiology*.

[30] Guzman-Beltran S., Perez-Torres A., Coronel-Cruz C., Torres-Guerrero H. (2012). Phagocytic receptors on macrophages distinguish between different *Sporothrix schenckii* morphotypes. *Microbes and Infection*.

[31] Boyce K. J., Andrianopoulos A. (2013). Morphogenetic circuity regulating growth and development in the dimorphic pathogen *Penicillium marneffei*. *Eukaryotic Cell*.

[32] Sterkel A. K., Mettelman R., Wüthrich M., Klein B. S. (2015). The unappreciated intracellular lifestyle of *Blastomyces dermatitidis*. *Journal of Immunology*.

[33] Brandhorst T. T., Roy R., Wüthrich M. (2013). Structure and function of a fungal adhesion that binds heparin and mimics thrombospondin-1 by blocking T cell activation and effector function. *PLoS Pathogens*.

[34] Beaussart A., Brandhorst T., Dufrêne Y. F., Klein B. S. (2015). *Blastomyces* virulence adhesion-1 protein binding to glycosaminoglycans is enhanced by protein disulfide isomerase. *MBio*.

[35] Brandhorst T., Wüthrich M., Finkel-Jimenez B., Klein B. S. (2003). A C-terminal EGF-like domain governs BAD1 localization to the yeast cell surface and fungal adherence to phagocytes, but is dispensable in immune modulation and pathogenicity of *Blastomyces dermatitidis*. *Molecular Microbiology*.

[36] Finkel-Jimenez B., Wüthrich M., Klein B. S. (2002). BAD1, an essential virulence factor of *Blastomyces dermatitidis*, suppresses host TNF-alpha production through TGF-beta-dependent and –independent mechanisms. *Journal of Immunology*.

[37] Finkel-Jimenez B., Wüthrich M., Brandhorst T. T., Klein B. S. (2001). The WI-1 adhesin blocks phagocyte TNF-alpha production, imparting pathogenicity on *Blastomyces dermatitidis*. *Journal of Immunology*.

[38] Brandhorst T. T., Wüthrich M., Finkel-Jimenez B., Warner T., Klein B. S. (2004). Exploiting type 3 complement receptor for TNF-alpha suppression, immune evasion, and progressive pulmonary infection. *Journal of Immunology*.

[39] FDA Alert (2008). *Tumor Necrosis Factor-Alpha Blockers (TNF Blockers), Cimzia (Certolizumab Pegol), Enbrel (Etanercept), Humira (Adalimumab), and Remicade (Infliximab)*.

[40] Brandhorst T. T., Wüthrich M., Warner T., Klein B. S. (1999). Targeted gene disruption reveals an adhesion indispensable for pathogenicity of *Blastomyces dermatitidis*. *Journal of Experimental Medicine*.

[41] Sterkel A. K., Lorenzini J. L., Fites J. S. (2016). Fungal mimicry of a mammalian aminopeptidase disables innate immunity and promotes pathogenicity. *Cell Host and Microbe*.

[42] Cooper K. G., Zarnowski R., Woods J. P. (2009). *Histoplasma capsulatum* encodes a dipeptidyl peptidase active against the mammalian immunoregulatory peptide, substance P. *PLoS One*.

[43] Cooper K. G., Woods J. P. (2009). Secreted dipeptidyl peptidase IV activity in the dimorphic fungal pathogen *Histoplasma capsulatum*. *Infection and Immunity*.

[44] Hung C. Y., Yu J. J., Seshan K. R., Reichard U., Cole G. T. (2002). A parasite phase-specific adhesion of *Coccidioides immitis* contributes to the virulence of this respiratory fungal pathogen. *Infection and Immunity*.

[45] Sebghati T. S., Engle J. T., Goldman W. E. (2000). Intracellular parasitism by *Histoplasma capsulatum*: fungal virulence and calcium dependence. *Science*.

[46] Isaac D. T., Berkes C. A., English B. C. (2015). Macrophage cell death and transcriptional response are actively triggered by the fungal virulence factor Cbp1 during *H. capsulatum* infection. *Molecular Microbiology*.

[47] Beck M. R., DeKoster G. T., Hambly D. M., Gross M. L., Cistola D. P., Goldman W. E. (2008). Structural features responsible for the biological stability of *Histoplasma’s* virulence factor CBP. *Biochemistry*.

[48] Beck M. R., DeKoster G. T., Cistola D. P., Goldman W. E. (2009). NMR structure of fungal virulence factor reveals structural homology with mammalian saposin. *Molecular Microbiology*.

[49] Bohse M. L., Woods J. P. (2007). RNA interference-mediated silencing of the YPS3 gene of *Histoplasma capsulatum* reveals virulence defects. *Infection and Immunity*.

[50] Kanetsuna F., Carbonell L. M. (1971). Cell wall composition of the yeastlike and mycelial forms of *Blastomyces dermatitidis*. *Journal of Bacteriology*.

[51] Kanetsuna F., Carbonell L. M., Moreno R. E., Rodriguez J. (1969). Cell wall composition of the yeast and mycelial forms of *Paracoccidioides brasiliensis*. *Journal of Bacteriology*.

[52] Koneti A., Linke M. J., Brummer E., Stevens D. A. (2008). Evasion of innate immune responses: evidence for mannose binding lectin inhibition of tumor necrosis factor alpha production by macrophages in response to *Blastomyces dermatitidis*. *Infection and Immunity*.

[53] Rappleye C. A., Eissenberg L. G., Goldman W. E. (2007). *Histoplasma capsulatum* alpha-(1,3)-glucan blocks innate immune recognition by the beta-glucan receptor. *Proceedings of the National Academy of Sciences, USA*.

[54] Klein B. S., Vergeront J. M., Weeks R. J. (1986). Isolation of *Blastomyces dermatitidis* from soil associated with a large outbreak of blastomycosis in Wisconsin. *New England Journal of Medicine*.

[55] Klein B. S., Vergeront J. M., DiSalvo A. F., Kaufman L., Davis J. P. (1987). Two outbreaks of blastomycosis along rivers in Wisconsin. Isolation of *Blastomyces dermatitidis* from riverbank soil and evidence of its transmission along waterways. *American Review of Respiratory Diseases*.

[56] Deepe G. S., Bennett J. E., Dolin R., Blaser M. J. (2015). Chapter 265–“*Histoplasma capsulatum* (histoplasmosis)”. *Mandell, Douglas, and Bennett’s Principles and Practice of Infectious Diseases*.

[57] Galgiani J. N., Bennett J. E., Dolin R., Blaser M. J. (2015). Chapter 267–“Coccidioidomycosis (*Coccidioides* species)”. *Mandell, Douglas, and Bennett’s Principles and Practice of Infectious Diseases*.

[58] Parente J. A., Borges C. L., Pereira M., Sullivan D. J., Moran G. P. (2014). *Paracoccidioides* mechanisms of pathogenesis and virulence. *Human Pathogenic Fungi Molecular Biology and Pathogenic Mechanisms*.

[59] Tavares A. H., Derengowski L. S., Ferreira K. S. (2012). Murine dendritic cells transcriptional modulation upon *Paracoccidioides brasiliensis* infection. *PLoS Neglected Tropical Diseases*.

[60] Drummond R. A., Brown G. D. (2011). The role of Dectin-1 in the host defense against fungal infections. *Current Opinion in Microbiology*.

[61] Silva S. S., Tavares A. H. F. P., Passos-Silva D. G. (2008). Transcriptional response of murine macrophages upon infection with opsonized *Paracoccidioides brasiliensis* yeast cells. *Microbes and Infection*.

[62] Nemecek J. C., Wüthrich M., Klein B. S. (2016). Global control of dimorphism and virulence in fungi. *Science*.

[63] Boyce K. J., Schreider L., Kirszenblat L., Andrianopoulos A. (2011). The two-component histidine kinases DrkA and SlnA are required for *in vivo* growth in the human pathogen *Penicillium marneffei*. *Molecular Microbiology*.

[64] Hou B., Zhang Z., Zheng F., Liu X. (2013). Molecular cloning, characterization, and differential expression of *DRK1* in *Sporothrix schenckii*. *International Journal of Molecular Medicine*.

[65] Chaves A. F., Navarro M. V., Castilho D. G., Calado J. C., Conceição P. M., Batista W. L. (2016). A conserved dimorphism-regulating histidine kinase controls the dimorphic switching in *Paracoccidioides brasiliensis*. *FEMS Yeast Research*.

[66] Nguyen N. Q., Sil A. (2008). Temperature-induced switch to the pathogenic yeast form of *Histoplasma capsulatum* requires Ryp1, a conserved transcriptional regulator. *Proceeding of the National Academy of Sciences, USA*.

[67] Webster R. H., Sil A. (2008). Conserved factors Ryp2 and Ryp3 control cell morphology and infectious spore formation in the fungal pathogen *Histoplasma capsulatum*. *Proceeding of the National Academy of Sciences, USA*.

[68] Beyham S., Gutierrez M., Voorhies M., Sil A. (2013). A temperature-responsive network links cell shape and virulence traits in a primary fungal pathogen. *PLoS Biology*.

[69] Gauthier G. M., Sullivan T. D., Gallardo S. S. (2010). SREB, a GATA transcription factor that directs disparate fates in *Blastomyces dermatitidis* including morphogenesis and siderophore biosynthesis. *PLoS Pathogens*.

[70] Marty A. J., Broman A. T., Zarnowski R. (2015). Fungal morphology, iron homeostasis, and lipid metabolism regulated by a GATA transcription factor in *Blastomyces dermatitidis*. *PLoS Pathogens*.

[71] Hwang L. H., Seth E., Gilmore S. A., Sil A. (2012). SRE1 regulates iron-dependent and –independent pathways in the fungal pathogen *Histoplasma capsulatum*. *Eukaryotic Cell*.

[72] Jung W. H., Sham A., White R., Kronstad J. W. (2006). Iron regulation of the major virulence factors in the AIDS-associated pathogen *Cryptococcus neoformans*. *PLoS Biology*.

[73] Hilty J., Smulian A. G., Newman S. L. (2008). The *Histoplasma capsulatum* vacuolar ATPase is required for iron homeostasis, intracellular replication in macrophages, and virulence in a murine model of histoplasmosis. *Molecular Microbiology*.

[74] Bugeja H. E., Hynes M. J., Andrianopoulos A. (2013). HgrA is necessary and sufficient to drive hyphal growth in the dimorphic pathogen *Penicillium marneffei*. *Molecular Microbiology*.

[75] Todd R. B., Greenhalgh J. R., Hynes M. J., Adrianopoulos A. (2003). TupA, the *Penicillium marneffei* Tup1p homologue, represses both yeast and spore development. *Molecular Microbiology*.

[76] Gilmore S. A., Naseem S., Konopka J. B., Sil A. (2013). N-acetylglucosamine (GlcNAc) triggers a rapid, temperature-responsive morphogenetic program in thermally dimorphic fungi. *PLoS Genetics*.

[77] Marty A. J., Wüthrich M., Carmen J. C. (2013). Isolation of *Blastomyces dermatitidis* yeast from lung tissue during murine infection for *in vivo* transcriptional profiling. *Fungal Genetics and Biology*.

[78] Muñoz J. F., Gauthier G. M., Desjardins C. A. (2015). The dynamic genome and transcriptome of the human fungal pathogen *Blastomyces* and close relative *Emmonsia*. *PLoS Genetics*.

[79] Citiulo F., Jacobsen I. D., Miramón P. (2012). *Candida albicans* scavenges host zinc via Pra1 during endothelial invasion. *PLoS Pathogens*.

[80] Wise H. Z., Hung C. Y., Whiston E., Taylor J. W., Cole G. T. (2013). Extracellular ammonia at sites of pulmonary infection with *Coccidioides posadasii* contributes to severity of the respiratory disease. *Microbial Pathogenesis*.

[81] Mirbod-Donovan F., Schaller R., Hung C. Y., Xue J., Reichard U., Cole G. T. (2006). Urease produced by *Coccidioides posadasii* contributes to the virulence of this respiratory pathogen. *Infection and Immunity*.

[82] Singh A., Panting R. J., Varma A. (2013). Factors required for activation of urease as a virulence determinant in *Cryptococcus neoformans*. *MBio*.

[83] Feder V., Kmetzsch L., Staats C. C. (2015). *Cryptococcus gattii* urease as a virulence factor and the relevance of enzymatic activity in cryptococcosis pathogenesis. *The FEBS Journal*.

[84] Chen S. C., Meyer W., Sorrell T. C. (2014). *Cryptococcus gattii* infections. *Clinical Microbiology Reviews*.

[85] Boyce K. J., McLauchlan A., Schreider L., Andrianopoulos A. (2015). Intracellular growth is dependent on tyrosine catabolism in the dimorphic fungal pathogen *Penicillium marneffei*. *PLoS Pathogens*.

[86] Nunes L. R., Costa de Oliveira R., D.B. Leite DB, et al. (2005). Transcriptome analysis of *Paracoccidioides brasiliensis* cells undergoing mycelium-to-yeast transition. *Eukaryotic Cell*.

[87] Uyttenhove C., Pilotte L., Théate I. (2003). Evidence for a tumoral immune resistance mechanism based on tryptophan degradation by indoleamine 2,3-dioxygenase. *Nature Medicine*.

[88] Hage C. A., Horan D. J., Durkin M. (2013). *Histoplasma capsulatum* preferentially induces IDO in the lung. *Medical Mycology*.

[89] Araújo E. F., Loures F. V., Bazan S. B. (2014). Indoleamine 2,3-dioxygenase controls fungal loads and immunity in Paracoccidioidomycosis but is more important to susceptible than resistant hosts. *PLoS Neglected Tropical Diseases*.

[90] Hennicke F., Grumbt M., Lermann U. (2013). Factors supporting cysteine tolerance and sulfite production in *Candida albicans*. *Eukaryotic Cell*.

[91] Grumbt M., Monod M., Yamada T., Hertweck C., Kunert J., Staib P. (2013). Keratin degradation by dermatophytes relies on cysteine dioxygenase and a sulfite efflux pump. *Journal of Investigative Dermatology*.

